# Long-term trends in incidence, mortality and burden of liver cancer due to specific etiologies in Hubei Province

**DOI:** 10.1038/s41598-024-53812-8

**Published:** 2024-02-28

**Authors:** Hao Liu, Jun Li, Shijie Zhu, Xupeng Zhang, Faxue Zhang, Xiaowei Zhang, Gaichan Zhao, Wei Zhu, Fang Zhou

**Affiliations:** 1 Institute of Chronic Disease Prevention and Cure, Hubei Provincial Center for Disease Control and Prevention, Wuhan, 430079 China; 2 Institute of Health Inspection and Testing, Hubei Provincial Center for Disease Control and Prevention, Wuhan, 430079 China; 3 Department of Occupational and Environmental Health, School of Public Health, Wuhan University, Wuhan, 430071 China; 4 Wuhan Changjiang New Area Center for Disease Control and Prevention, Wuhan, 430345 China; 5 Department of Public Health, School of Public Health, Wuhan University, Wuhan, 430071 China

**Keywords:** Age–period–cohort analysis, Liver cancer, Incidence, Mortality, Hepatitis virus, Cancer, Cancer epidemiology, Gastrointestinal cancer

## Abstract

Liver cancer, a chronic non-communicable disease, represents a serious public health problem. Long-term trends in the burden of liver cancer disease are heterogeneous across regions. Incidence and mortality of liver cancer, based on the Global Burden of Disease, were collected from the Chinese Centre for Disease Control and Prevention. Age–period–cohort model was utilized to reveal the secular trends and estimate the age, period and cohort effects on primary liver cancer due to specific etiologies. Both the age-standardized incidence and mortality rate of liver cancer in Hubei province were on the rise, although there were discrepancies between gender groups. From age–period–cohort analysis, both incidence and mortality of liver cancer due to Hepatitis B virus were the highest in all age groups. The incidence of all liver cancer groups increased with time period in males, while this upward trend was observed in females only in liver cancer due to alcohol use group. Cohort effects indicated the disease burden of liver cancer decreased with birth cohorts. Local drifts showed that the incidence of liver cancer due to specific etiologies was increasing in the age group of males between 40 and 75 years old. The impact of an aging population will continue in Hubei Province. the disease burden of liver cancer will continue to increase, and personalized prevention policies must be adopted to address these changes.

## Introduction

Liver cancer is becoming a growing public health concern worldwide^1^. China is a country with a high incidence of liver cancer, and the burden of liver cancer in China has been among the highest around the world, with chronic infection with hepatitis B virus (HBV) as the main cause of primary liver cancer in the population^2^.

In 2017, according to the GBD-China report, a comparison of provincial age-standardized years of life lost (YLLs) for the top 20 causes of disease in China revealed that Hubei Province had a higher age-standardized YLL rate for liver cancer (660 person-years/100,000 population) compared to the national average (550 years/100,000 population), ranked fifth among all provincial administrative regions^3^. However, the existing research evidence is relatively outdated and consists of individual annual reports, lacking systematic trend analysis and etiological reports. The main histological type of liver cancer is hepatocellular carcinoma (HCC), which accounts for approximately 75% of all liver cancer cases^4–6^. HBV, hepatitis C virus (HCV), high body mass index (BMI) and aflatoxins are partial risk factors for primary liver cancer^7–9^. Although many risk factors lead to liver cancer, the incidence of liver cancer due to HBV, HCV and alcohol consumption accounts for over 90% of the total liver cancer incidence in China^10^.

In recent years, with the rapid development of land transportation and the acceleration of population mobility, risk factors influencing diseases have undergone rapid changes across different regions. A study on the concentration of health resources suggests that enhancing the radiating effect of core provincial (municipal) health resources, rationalizing the allocation of health resources, and shifting perspectives to support policies on public health resource services are beneficial for alleviating the burden of diseases^11^. As the central province in the Central China region, Hubei serves as a key transportation hub in China with the rapid development of road transportation. According to the 2021 National Population Census of China, Hubei Province ranks tenth in the country with 12.47 million immigrants^12^. While some studies have described the incidence or mortality rates of cancer in the Hubei region, there has not been research on the long-term disease patterns and specific causative changes in cancer in Hubei Province^13,14^. It is essential to study the long-term trends in cancer incidence and mortality rates in this region to reveal the patterns of cancer occurrence, provide supportive information for policy formulation and adjustments, facilitate the rational allocation of health resources, and reduce the disease burden of cancer.

Age–period–cohort (APC) models were commonly used to study age, period and birth cohort effects on disease^15^. In this study, the APC model was performed to explore the disease burden of primary liver cancer caused by specific etiologies in Hubei Province from 1990 to 2019, with a view to reveal changes and provide a scientific basis for the adjustment of liver cancer prevention and control policies.

## Materials and methods

### Data resources

The original data was part of the Global Burden of Disease and collected through a request to the Life Registration and Cause of Death Surveillance Unit of the Chinese Centre for Disease Control and Prevention (CDC). The incidence and death rate of liver cancer due to hepatitis B, hepatitis C and other causes in children aged under 5 years, as well as liver cancer due to alcohol consumption in children aged 0–14 years are very low (close to 0), so the data has been excluded from the following analysis.

### Definition of disease

The specific causes of primary liver cancer in this study included hepatitis B, hepatitis C, alcohol consumption and other causes. The International Classification of Diseases (ICD-10) criteria was used to define primary liver cancer and the various specific causes of liver cancer (C22.0–C22.4, C22.7–C22.9).

### Age–period–cohort analysis

The APC framework was used to assess the incidence and mortality of liver cancer in three effect dimensions: age, period and cohort^16^. As periods and age intervals in the APC tool should be fixed and equal^17^, the 95+ years group was further excluded from the analysis. The age effect reflects how individuals of different ages might experience different rates or risks of an event or condition. Period effect represents the influence of external factors that affect all age groups simultaneously during a specific period of time. It captures the effects of events or exposures that affect the entire population at a given period in history. The cohort effect, also known as the birth cohort effect, refers to the impact of shared experiences or exposures among individuals who were born during the same time period. The following functions reflect age, period and cohort effects for different aspects.

(1) Longitudinal age curve, age-specific morbidity/mortality of liver cancer adjusted for period deviation in the reference cohort, illustrates how the incidence/mortality of liver cancer changes over time across different ages while accounting for period and cohort effects. Examining the shape and trajectory of the curve can identify patterns and trends in the incidence/mortality of liver cancer across different age groups, periods, and cohorts. It helps distinguish whether observed changes are primarily due to aging, external factors affecting the entire population, or cohort-specific influences. (2) Period rate ratio (PRR), the ratio of age-specific incidence/mortality for each period relative to the reference period, is a measure used to quantify the effect of period changes on the incidence/mortality of liver cancer. (3) Fitted time trend shows the change in expected incidence/mortality over time for the reference age group adjusted for cohort effects. (4) Cohort rate ratio (CRR), the ratio of age-specific incidence/mortality for each cohort relative to the reference cohort, is a measure used to assess the impact of cohort-specific factors on the incidence/mortality of liver cancer. (5) Net drift refers to the overall or long-term change in the cohort-specific effects on an outcome variable over time and captures the average change in the incidence/mortality of liver cancer across all cohorts. (6) Local drifts (also known as age-specific estimated annual percentage changes) Local drifts capture variations around the net drift and highlight specific cohort groups that may be experiencing different trends compared to the overall cohort pattern. The reference age, period and cohort were set as the middle group of age, period, and cohort, respectively.

The above effects analysis can be used to obtain estimable parameters with the help of the APC web tool (Biometrics Branch, National Cancer Institute, Bethesda, Maryland https://analysistools.cancer.gov/apc/). The Wald chi-square test was used to investigate the statistical significance of the estimable function.

### Ethical approval

This study did not involve human participants. This study was approved for exemption by the Ethics Committee of the Wuhan University School of Public Health. All the data comes from secondary unidentifiable records. All methods were performed in accordance with the relevant guidelines and regulations.

### Consent to participate

The Ethics Committee of the Wuhan University School of Public Health confirmed that this study did not involve consent for participation.

## Results

Table 1 shows the incidence and mortality rates of liver cancer due to specific etiologies in Hubei Province, by gender, 1990–2019.

**Table 1:** Incidence and mortality rates of liver cancer due to specific etiologies in Hubei Province, by gender, 1990–2019.

	1990–1994						2015–2019					
	Male			Female			Male			Female		
	Number	CR	ASR	Number	CR	ASR	Number	CR	ASR	Number	CR	ASR
Incidence												
LCHB	4708	17.16	24.77	1689	6.52	9.12	13,038	46.48	35.85	2992	11.31	8.73
LCHC	414	1.51	2.53	209	0.81	1.29	1403	5.00	4.06	449	1.70	1.38
LCAU	227	0.83	1.26	1058	4.08	6.27	683	2.43	1.87	2494	9.43	7.35
LCOC	2840	10.35	15.21	197	0.76	1.07	7736	27.58	21.56	330	1.25	0.99
Total	8189	29.86	43.77	3153	12.17	17.74	22,860	81.49	63.33	6266	23.69	18.447
Mortality												
LCHB	4621	16.85	25.21	1612	6.22	9.09	9319	33.22	26.46	2311	8.74	6.87
LCHC	423	1.54	2.68	216	0.83	1.37	1045	3.73	3.16	370	1.40	1.17
LCAU	230	0.84	1.31	1100	4.24	6.63	496	1.77	1.40	2026	7.66	6.11
LCOC	2798	10.20	15.56	189	0.73	1.08	5579	19.89	16.10	259	0.98	0.79
Total	8072	29.43	44.76	3116	12.02	18.16	16,439	58.61	47.12	4966	18.78	14.95

*CR: Crude rate; ASR: Age-standardized rate; LCHB: Liver cancer due to hepatitis B virus; LCHC: Liver cancer due to hepatitis C virus; LCAU: Liver cancer due to alcohol use; LCOC: Liver cancer due to other causes.

### Incidence

In Hubei, age standardized incidence rates (ASIR) for liver cancer due to hepatitis B virus (LCHB), liver cancer due to hepatitis C virus (LCHC) and liver cancer due to other causes (LCOC) groups were higher in males than females, but higher in females than in males only in the liver cancer due to alcohol use (LCAU) group (Figure S1). The ASIR showed an increasing trend across liver cancer groups in male, but remained stable in all female liver cancer groups.

### Mortality

In Hubei, the risk of death from all types of liver cancer, except LCAU, was higher for males than females. In both male and female groups, the age-standardized mortality rates (ASMR) for liver cancer due to specific causes remained largely stable, showing a downward trend after reaching a peak in 2005, except for the LCAU in females.

### Age–period–cohort analysis

**Figure 1:**
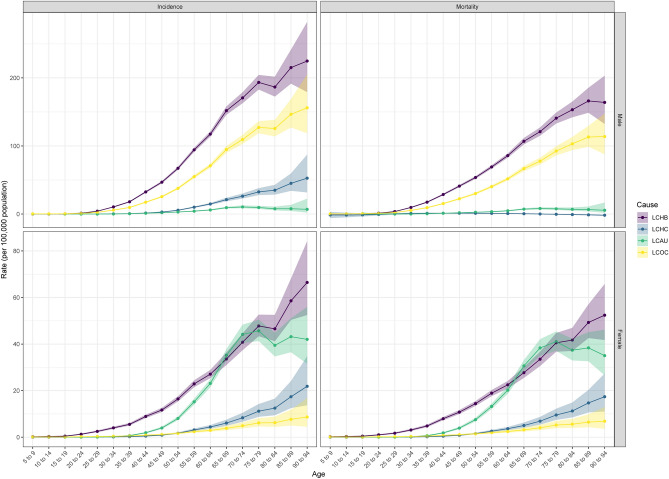
Longitudinal age curve of liver cancer due to specific etiologies incidence and mortality in males and females in Hubei.

The age incidence pattern of liver cancer is almost the same for LCHB, LCHC and LCOC, while liver cancer caused by alcohol use showed a decreasing trend after the age of 75–80, with incidence rates of LCHB > LCHC > LCAU > LCOC in all age groups (Fig. 1). The age-specific incidence of liver cancer in females also increased with age, with LCAU age-specific mortality rates being significantly higher after the 40–45 age group than in males and exceeding the incidence of LCHB in females of the same age group in 65–69 and 70–74. Age-specific mortality rates increased with age in males in Hubei Province, with LCHB > LCHC > LCAU > LCOC for each specific cause of liver cancer. Age-specific mortality rates for females also increased with age, with age-specific mortality rates for LCAU exceeding those for LCHB in three age groups: 65–69, 70–74 and 75–79 years. In the male group, the fitted time trend results for all liver cancer types showed an increasing trend in incidence, which could be observed in the LCAU group in females (Figure S3). As for mortality, all but the female LCAU group showed a downward trend after 2005.

**Figure 2:**
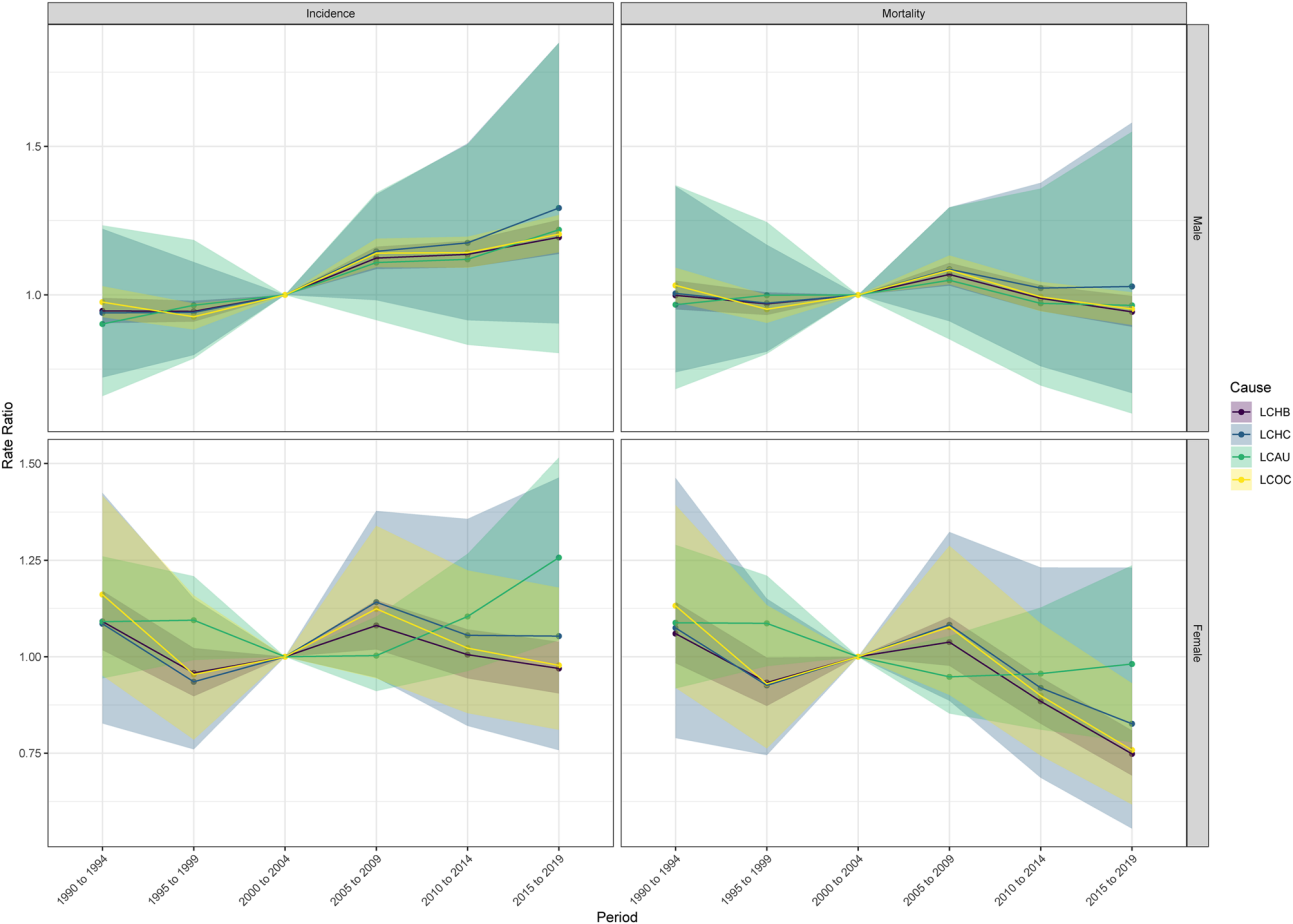
Period Rate Ratios of liver cancer due to specific etiologies incidence and mortality in males and females in Hubei.

The PRRs for males in Hubei province showed an increasing trend and were all > 1 after 2005 (Fig. 2). While the PRRs for females in Hubei province were significantly different from those for males, the PRRs for LCHB being > 1 in 1990–1994 and 2005–2009; the PRRs for LCHC being > 1 in all periods except for 1995–1999, when the PRRs was < 1; and the PRRs for LCAU being > 1 in all periods relative to the reference period, with a rapid increase after 2005. The PRR for LCOC was < 1 in 1995–1999 and 2015–2019 and > 1 in all other periods. For the Wald test for change in PRRs of incidence, the difference in PRRs between the male LCHC and LCAU groups and the female LCHC and LCOC groups was not significant (p > 0.05). The PRRs for male mortality relative to the reference period were > 1 for the LCHB from 2005–2009, and < 1 for all other periods, and > 1 for the LCHC after 2005, with an increasing trend. The Wald test indicated that the differences between the male LCHC and LCAU groups as well as the female LCHC and LCAU groups were not statistically significant (p > 0.05).

**Figure 3:**
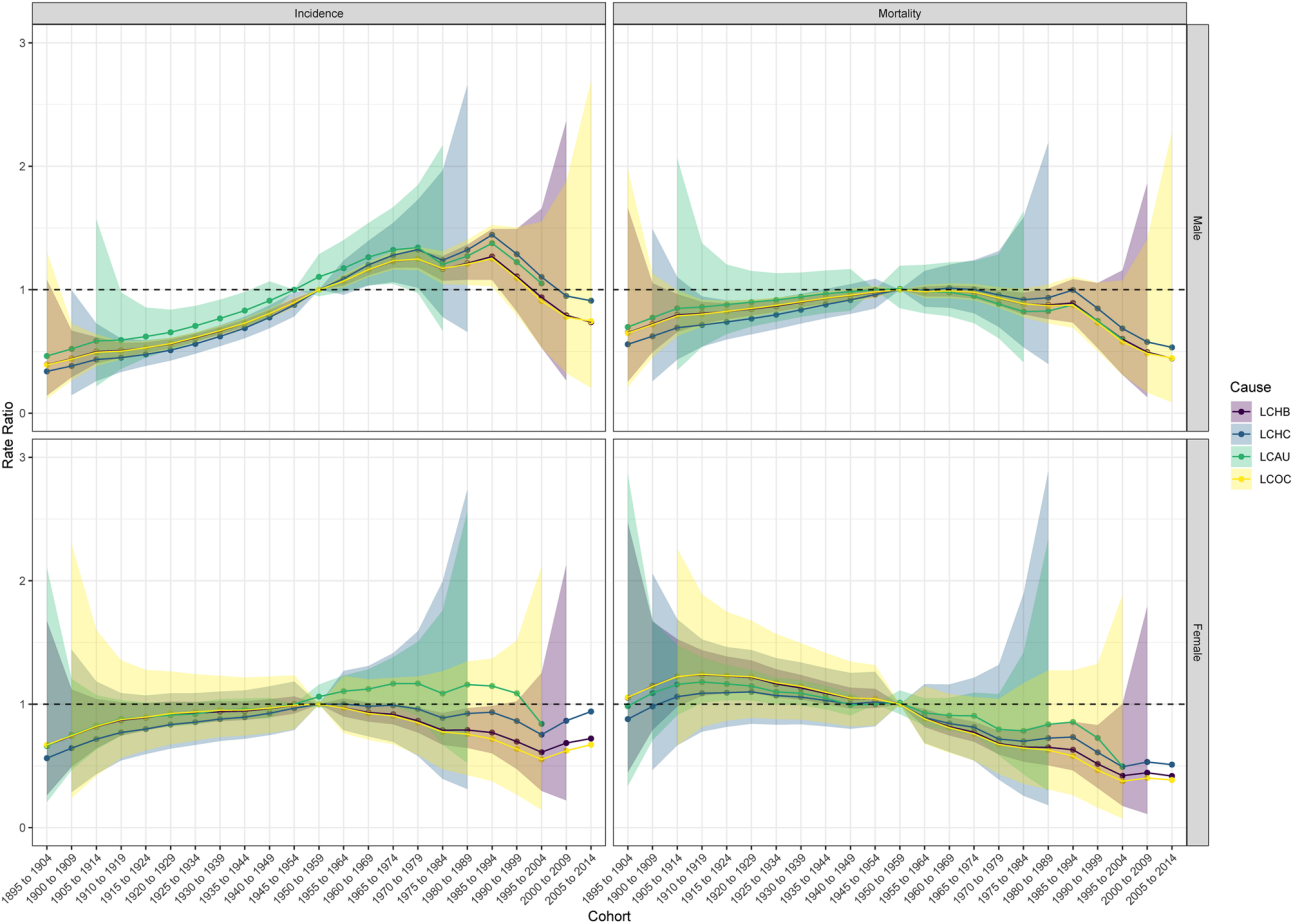
Cohort Rate Ratios of liver cancer due to specific etiologies incidence and mortality in males and females in Hubei.

The CRRs for the incidence of liver cancer of all causes in males in Hubei Province indicated a decreasing trend among those born after 1995, while the CRR for the incidence of liver cancer of all causes in females showed an increasing trend among those born after 2000 (except for LCAU) (Fig. 3). The Wald test for changes in incidence CRRs in both sexes showed statistically significant differences in the male liver cancer groups (p < 0.05) (Table S1). The CRRs for mortality from liver cancer of all causes in males showed a decreasing trend among those born after 1995, while mortality from liver cancer of all causes in females basically showed a decreasing trend among those born after 1955, with fluctuating decreases in the LCHC and LCAU groups. The Wald test for the change in CRRs for both genders showed a significant difference in both the LCHB group and the male LCOC group (p < 0.05).

The net drift revealed an increasing trend in the incidence of LCHB and LCOC in males and a decreasing trend in mortality in both LCHB and LCOC in females (Table 2).

**Table 2:**
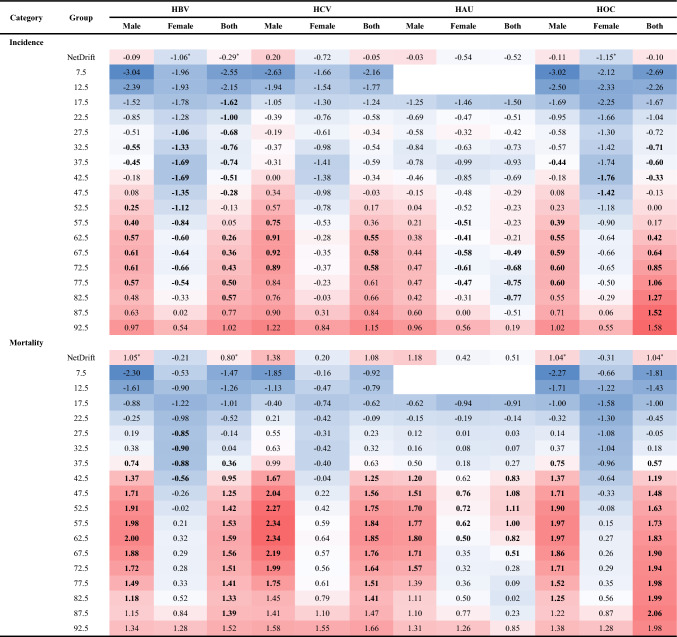
Net Drift and Local Drifts of liver cancer due to specific etiologies on incidence and mortality in males and females in Hubei.

* Note: Red refers to APC > 0; Blue refers to APC < 0. Bold refers to the result of wald test for the null hypothesis “Net Drift = 0” are not statistically significant, p < 0.05. “*” refers to the result of wald test for the null hypothesis “Local Drifts = Net Drift” are not statistically significant, p < 0.05.

Local drifts showed a decreasing trend in the incidence of LCHB in females in the 25–44 years age group. However, the incidence of LCAU in females tends to increase in the 45–64 years age group. The incidence of different types of liver cancer in males presented a significantly increasing trend in age groups above 40 years. In parallel, Mortality in males for LCHB, LCHC, and LCOC increased in the 55–74 years age group.

## Discussion

Our results showed that liver cancers due to specific etiologies have significantly different long-term trends between gender groups. The incidence and mortality of LCHB were both highest across all age groups in males, while were lower than LCAU in 65–69 years and 70–74 years age group in females in Hubei. In the female population in Hubei Province, LCAU ranked as the second-highest in both incidence and mortality rates among liver cancers. In males, LCOC ranked as the second-highest in both incidence and mortality rates among liver cancers. The burden of liver cancer in Hubei province was increasing against the backdrop of a declining overall liver cancer burden in China. Both the incidence and mortality rates of liver cancer are on the rise among males. In the female population, the incidence and mortality rates of liver cancer due to hepatitis B are on the decline. The risk, both incidence and mortality, of liver cancer due to specific etiologies was reduced in the newborn cohort. It was closely related to the aggressive liver cancer prevention and control policy in recent decades in China^18,19^. Besides, there was a significant increase in mortality from all types of liver cancer in people aged 40–79 years, especially in males.

Hepatitis B continues to stand as the most important risk factor contributing to the onset of liver cancer in Hubei Province, encompassing both male and female populations. Notably, liver cancer due to hepatitis B had a faster decline in ASIR and ASMR than liver cancer due to other etiologies, aligning with the vaccination policy embedded in the hepatitis B vaccination schedule implemented in China since 1992^20^. It is noteworthy that a discernible trend in the escalation of liver cancer cases and ASIR for various causative factors has been observed in Hubei Province. This suggests a limited impact of certain broad-level prevention policies aimed at mitigating liver cancer. From 1990 to 2019, the ASIR for LCHB showed an increasing trend in males, surpassing the average level of LCHB in females. It has been suggested that there may be a super-additive and super-multiplicative interaction between the family history of liver cancer and HBV infection in the development of liver cancer^21^. The hepatitis B vaccine has a protection period of at least 12 years, with antibody levels decreasing over time, and issues of incomplete vaccination against hepatitis B persist^22,23^. With advancing age, the concentration of hepatitis B antibodies in the human body gradually decreases. This phenomenon significantly contributes to the notable decline in hepatitis B incidence among children in recent years, while incidence rates among the adult population remain relatively stable^24^. The administration of the hepatitis B booster shot is not mandatory, and there is limited willingness among individuals to receive it before knowing that their hepatitis B antibody test results are negative. Additionally, the detection rate of hepatitis B antigen negativity in population health check-ups tends to be higher in the elderly compared to the younger population, as older individuals undergo health check-ups more frequently. This presents a practical challenge in addressing the disease burden of liver cancer due to hepatitis B.

Although LCHC was apparently lower than LCHB at ASIR levels, LCHC remained a serious public health problem in developing countries^25–27^. The application of direct-acting antiviral drugs (DAAs) for hepatitis C treatment boasts a cure rate exceeding 90%^28^. In this study, the trend in the incidence of LCHC was generally consistent with that of hepatitis B. Despite the implementation of efficacious treatment protocols, the ASIR for LCHC remains elevated in Hubei Province. This can be attributed to the often-asymptomatic nature of acute hepatitis C, rendering it challenging for patients to detect. Consequently, there is an increased likelihood of its progression to chronic hepatitis C, culminating in the development of LCHC^29^. Considering that the transmission route of hepatitis C partially intersects with that of hepatitis B, coupled with the absence of an effective vaccine for HCV, it is recommended to combine screening for hepatitis B to reduce the financial cost while increasing the detection rate of acute and chronic hepatitis C for early detection, diagnosis and treatment^30^.

Alcohol consumption is strongly associated with the development of liver cancer, a phenomenon that is more common in males^31^. The incidence and mortality rates of LCAU in Hubei Province exhibited noteworthy disparities from those observed at the national level in China, particularly when considering distinctions between male and female groups. According to the analysis of alcohol consumption in Chinese patients with liver disease, males are usually the main consumers of alcohol and both ASIR and ASMR are higher in males than in females at the overall level in China^32^. The underlying reasons for this specific pattern in Hubei Province may be attributed to the relatively lower alcohol tolerance in the female population, the consumption of specific types of alcoholic beverages, and the presence of certain mutagens that heighten the susceptibility of females to alcohol^33–35^. Consequently, further research is imperative to elucidate the distinctive trend observed in Hubei Province.

LCOC is the second most common liver cancer in males in Hubei Province, with other causes including non-alcoholic diet, aflatoxins, tobacco and obesity^36,37^. The ASIR for liver cancer due to other causes remained largely unchanged in the Hubei Province female population, which may be related to the acquisition of risk factors. The gender specificity of hepatocarcinogenesis was revealed in a study on the association of metabolic syndrome (MetS) and its components with liver cancer^38^. In a prospective cohort study, the results indicate that the significant risk factors associated with an increased incidence of liver cancer in men are central obesity and hyperglycemia^7,38^. In studies of sex hormones, higher androgen receptor density is associated with increased visceral fat, which is more important than total obesity in the carcinogenic role of the liver^39,40^. There was another study reporting on the role of sex hormone signaling in the mechanism between type 2 diabetes and hepatocarcinogenesis^41^. These facts provided strong evidence for differences in metabolism-related factors between gender groups. The higher availability of these risk factors in males than in females accounts for the long-standing higher overall incidence of liver cancer in males than in females.

According to the results of the seventh population census, the proportion of people aged 60 + years in the resident population in Hubei Province was 20.42%, up 6.49 percentage points from 2010. Among the population aged 60 and above in Hubei Province, 6.6380 million people aged 60–69 are in the lower age group, accounting for 56.28% of the total elderly population, 0.45 percentage points higher than the national average, and the degree of aging has deepened^42^. The changes in the age structure of the population lead to changes in the burden of disease^43^. The age profile of incidence and mortality of LCAU in females in Hubei Province showed significant specificity. Both incidence and mortality rates of LCAU increase at a significantly higher rate with age than other types of liver cancer, and surpass LCHB as the type of liver cancer with the most serious disease burden in the region in the age groups of 65–69 and 70–74 years. This evidence should be considered when making adjustments to liver cancer prevention and control policies in Hubei Province.

The period covered by this study was one in which Chinese economic and social development had achieved remarkable results after more than 10 years of Reform and Openness, and where living standards and medical facilities and services had continued to improve. In our research, the incidence of liver cancer in males in Hubei Province showed a continuous upward trend with the change of period, and the risk of liver cancer from various causes did not decrease with the improvement of economic conditions and medical technology compared with the reference period. Studies in recent years have shown that economic and social development is positively correlated with the prevalence of chronic diseases and negatively correlated with acute infectious diseases^44^. Therefore, we believe that the economic and social development in Hubei Province has limited the progression of liver cancer due to viral hepatitis to a certain extent, but the significant increase in material conditions, and the high-energy diet leading to obesity and diabetes are generally high in the male population, and these diseases are associated with the development of liver cancer^37^. In addition, alcohol consumption and high energy diet are not uncommon in the group of patients with viral hepatitis, and the presence of the former is detrimental to the prognosis of acute viral hepatitis and increases the likelihood of acute viral hepatitis turning into chronic viral hepatitis^45^, leading to an increased incidence of LCHB and LCHC. In the study of Wang et.al, it was suggested that improvements in medical conditions have increased the screening rate of liver cancer^18^. Not only improvements in medical technology, but also social development, have led to concern for the health of the population^46,47^, which increased the screening rate of liver cancer. A general decrease in mortality rates for all types of liver cancer was shown after 2010, suggesting that improved medical technology has reduced the risk of death from liver cancer.

In China, the period before 1949 was one of instability and extreme lack of living, material and medical conditions, which led to the slow development of public health in China ^48^. After the founding of the People's Republic of China, there was a lack of a solid industrial foundation, coupled with natural disasters that further caused famine, and it was only after the reform and opening up that the economy entered a phase of steady and rapid growth. To set the reference cohorts for LCHB, LCHC and LCOC in 1955 and for LCAU in 1950 is a reasonable choice, taking into account the facts of the above historical process. We assumed that medical investment and development will also be hampered and the risk of liver cancer incidence and mortality will rise when the general economic and social development of China encounters setbacks. In this study, the birth cohort before the reference cohort demonstrated a gradual increase in their risk of liver cancer incidence and death. In the male group, the turning point in the downward trend in the risk of death for the birth cohort occurs after the reference cohort, when the risk of death from liver cancer declines rapidly after a short rise in the birth cohort. In the female cohort, the risk of death from liver cancer has been declining since the birth cohort after 1915, and plateaued after 2000. The effect of the cohort on liver cancer incidence and mortality is largely consistent with previous study^18^. For example, after the introduction of hepatitis B vaccination in newborns in China in 1992, the risk of LCHB incidence and mortality declined dramatically in men and women in Hubei Province, but we also note that in recent years this downward trend has not only slowed in the female population in Hubei Province, but has even increased in incidence. Further prevention and control measures need to be considered.

Some strengths of this study need to be claimed. First, the Age–Period–Cohort model is a classic model for studying the burden of disease and can provide valuable insight into long-term trends in disease. Secondly, the source of the data is mainly retrieved from the cancer registries, and birth and death registration systems in the following parts of Hubei Province. Finally, we reveal differences in the pattern of liver cancer disease due to different etiologies. Nevertheless, certain limitations characterize the present investigation. Firstly, the data on the disease burden of liver cancer in Hubei Province are all five-year averages, which leads to a reduction in the precision of our study. Secondly, some of the risk factors for liver cancer were not further differentiated and were included in the vague "other" subgroup. Thirdly, risk factors for liver cancer have a mutually reinforcing effect on each other, but this information was not refined during data collection and we were unable to analyze these factors further in this study. Finally, this research is a descriptive study with relatively insufficient ability to reveal the causes of trends.

### Supplementary Information

Supplementary Information.

## Data Availability

The corresponding author can be contacted at zhoufang2022@163.com for further clarification if required.

## Abbreviations

APC: Age–period–cohort
ASIR: Age-standardized incidence rate
ASMR: Age-standardized mortality rate
LCHB: Liver cancer due to hepatitis B
LCHC: Liver cancer due to hepatitis C
LCAU: Liver cancer due to alcohol use
LCOC: Liver cancer due to other cause
HCC: Hepatocellular carcinoma
YLL: Years of life lost
HBV: Hepatitis B virus
HCV: Hepatitis C virus
