# Supplementary material for: Long-term trends in incidence, mortality and burden of liver cancer due to specific etiologies in Hubei Province
Source: Sci Rep. 2024 Feb 28;14:4924. doi: 10.1038/s41598-024-53812-8 (PMC10902496; doi:10.1038/s41598-024-53812-8)
Supplement: Supplementary file 1 — Supplementary Information. [file 41598_2024_53812_MOESM1_ESM.docx]

**Supplementary Materials**

**1. Major abbreviation**

APC: Age-period-cohort

ASIR: Age-standardized incidence rate

ASMR: Age-standardized Mortality rate

CIR: Crude incidence rate

CMR: Crude mortality rate

LCHB: Liver cancer due to hepatitis B

LCHC: Liver cancer due to hepatitis C

LCAU: Liver cancer due to alcohol use

LCOC: Liver cancer due to other cause

**2.eMethod**

The partial null and alternative hypotheses associated with the tests in our analyses were shown in the following table.

| **Null Hypothesis** | **Implications** | **Degrees of Freedom** |
| --- | --- | --- |
| Net drift = 0 | Fitted temporal trends are stable (i.e., flat with no change) over time. Fitted longitudinal and cross-sectional age curves are proportional. | 1 |
| All period rate ratios = 1 | Net drift is 0 and fitted temporal trends are constant; Cross-sectional age curve describes age incidence pattern in every period. | *P* - 1 |
| All cohort rate ratios = 1 | Net drift is 0 and all local drifts are 0; Longitudinal age curve describes age incidence pattern in every cohort. | C - 1 |
| All local drifts = the net drift | Temporal patterns are the same in every age group. | *A-1* if *A=P*, *A* otherwise |

*For APC model defined over *A* (age groups), *P* (calendar periods), and *C* (birth cohorts) *= P + A – 1*.

Table S1 Wald Chi-Square tests for estimable functions in the APC model.

| Group |  | Incidence |  | Mortality | |
| --- | --- | --- | --- | --- | --- |
| **LCHB male** |  | **All PRR = 1** | **All CRR = 1** | **All PRR = 1** | **All CRR = 1** |
|  | χ^2^ | 82.78 | 899.91 | 66.00 | 65.67 |
|  | df | 5 | 22 | 5 | 22 |
|  | P-Value | **＜0.05*** | **＜0.05*** | **＜0.05*** | **＜0.05*** |
| **LCHB female** |  |  |  |  |  |
|  | χ^2^ | 28.84 | 21.89 | 106.24 | 85.88 |
|  | df | 5 | 22 | 5 | 22 |
|  | P-Value | **＜0.05*** | 0.47 | **＜0.05*** | **＜0.05*** |
| **LCHC male** |  |  |  |  |  |
|  | χ^2^ | 4.44 | 124.22 | 3.70 | 16.84 |
|  | df | 5 | 22 | 5 | 22 |
|  | P-Value | 0.49 | **＜0.05*** | 0.59 | 0.77 |
| **LCHC female** |  |  |  |  |  |
|  | χ^2^ | 5.75 | 4.67 | 10.00 | 3.93 |
|  | df | 5 | 22 | 5 | 22 |
|  | P-Value | 0.33 | 1.00 | 0.07 | 1.00 |
| **LCAU male** |  |  |  |  |  |
|  | X2 | 1.47 | 39.46 | 1.88 | 2.38 |
|  | df | 5 | 20 | 5 | 20 |
|  | P-Value | 0.92 | **＜0.05*** | 0.87 | 1.00 |
| **LCAU female** |  |  |  |  |  |
|  | χ^2^ | 39.74 | 17.91 | 8.63 | 19.58 |
|  | df | 5 | 20 | 5 | 20 |
|  | P-Value | **＜0.05*** | 0.59 | 0.12 | 0.49 |
| **LCOC male** |  |  |  |  |  |
|  | χ^2^ | 90.38 | 531.84 | 51.47 | 41.70 |
|  | df | 5 | 22 | 5 | 22 |
|  | P-Value | **＜0.05*** | **＜0.05*** | **＜0.05*** | **＜0.05*** |
| **LCOC female** |  |  |  |  |  |
|  | χ^2^ | 6.77 | 3.17 | 17.33 | 10.28 |
|  | df | 5 | 22 | 5 | 22 |
|  | P-Value | 0.24 | 1.00 | **＜0.05*** | 0.98 |

Note: Bold and “*” results are statistically significant (p<0.05). PRR, period rate ratio; CRR, cohort rate ratio.


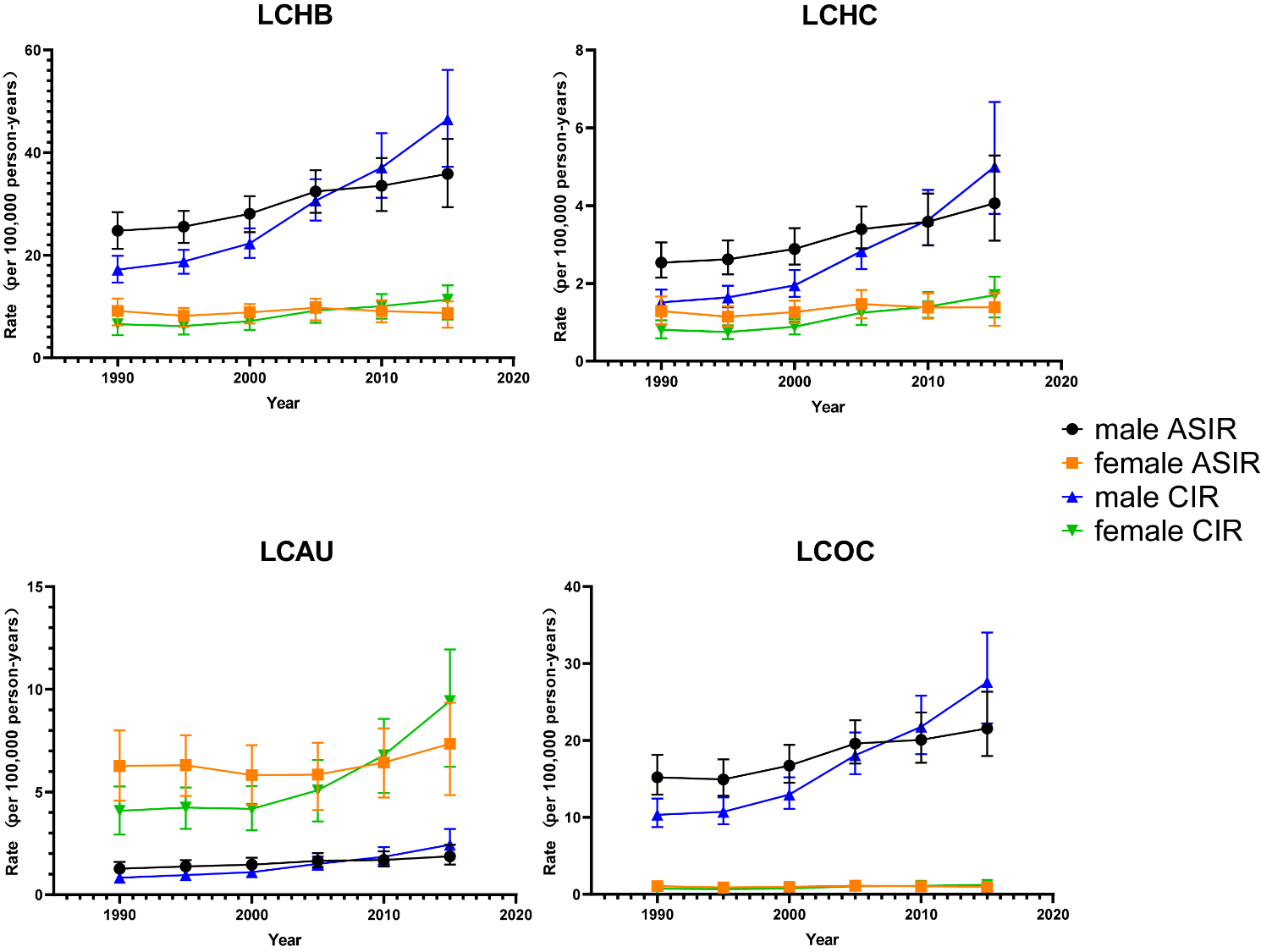


Figure S1. Incidence rates of liver cancer due to specific etiologies in Hubei Province, by gender, 1990-2019.


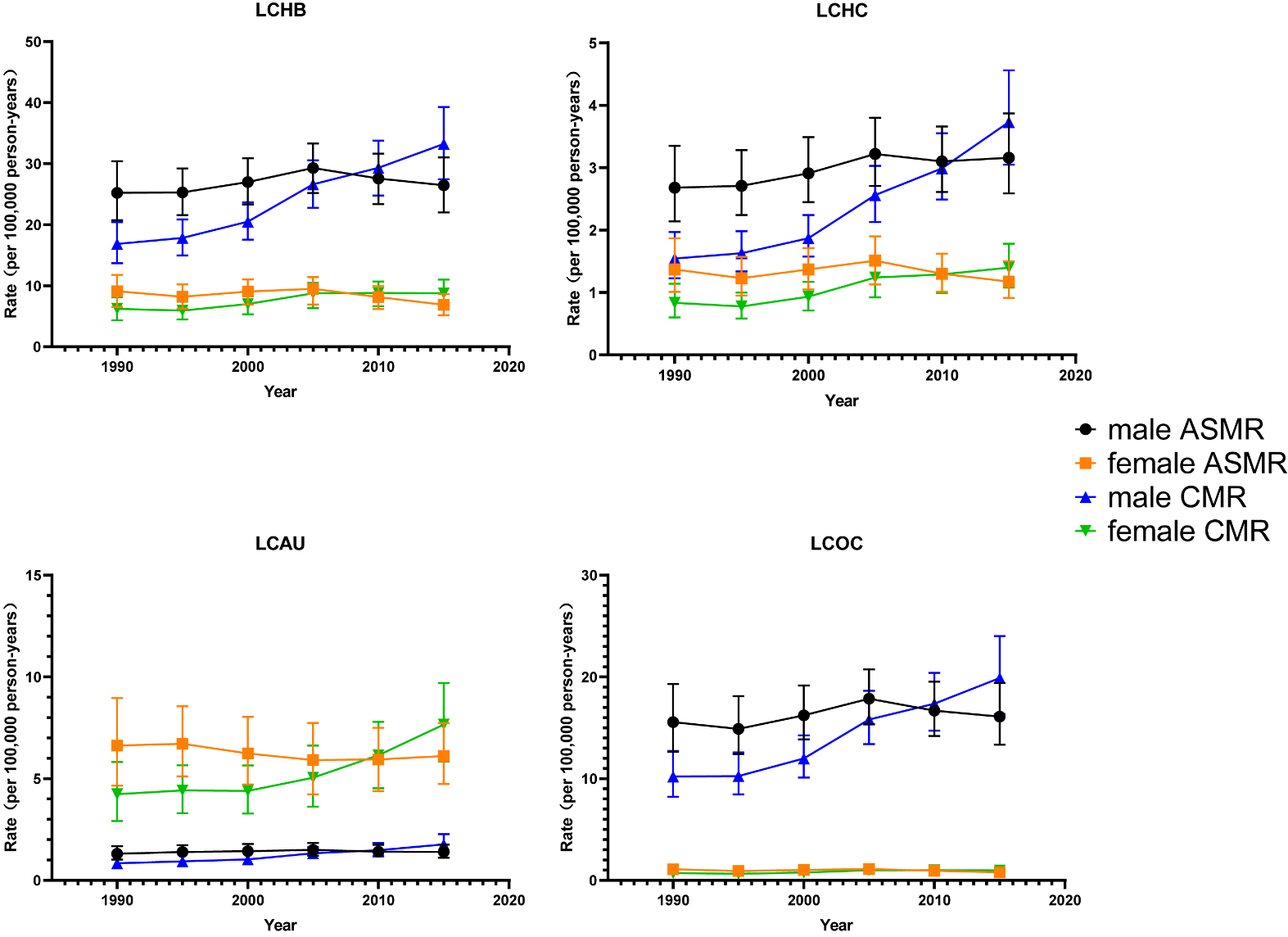


Figure S2. Mortality rates of liver cancer due to specific etiologies in Hubei Province, by gender, 1990-2019.


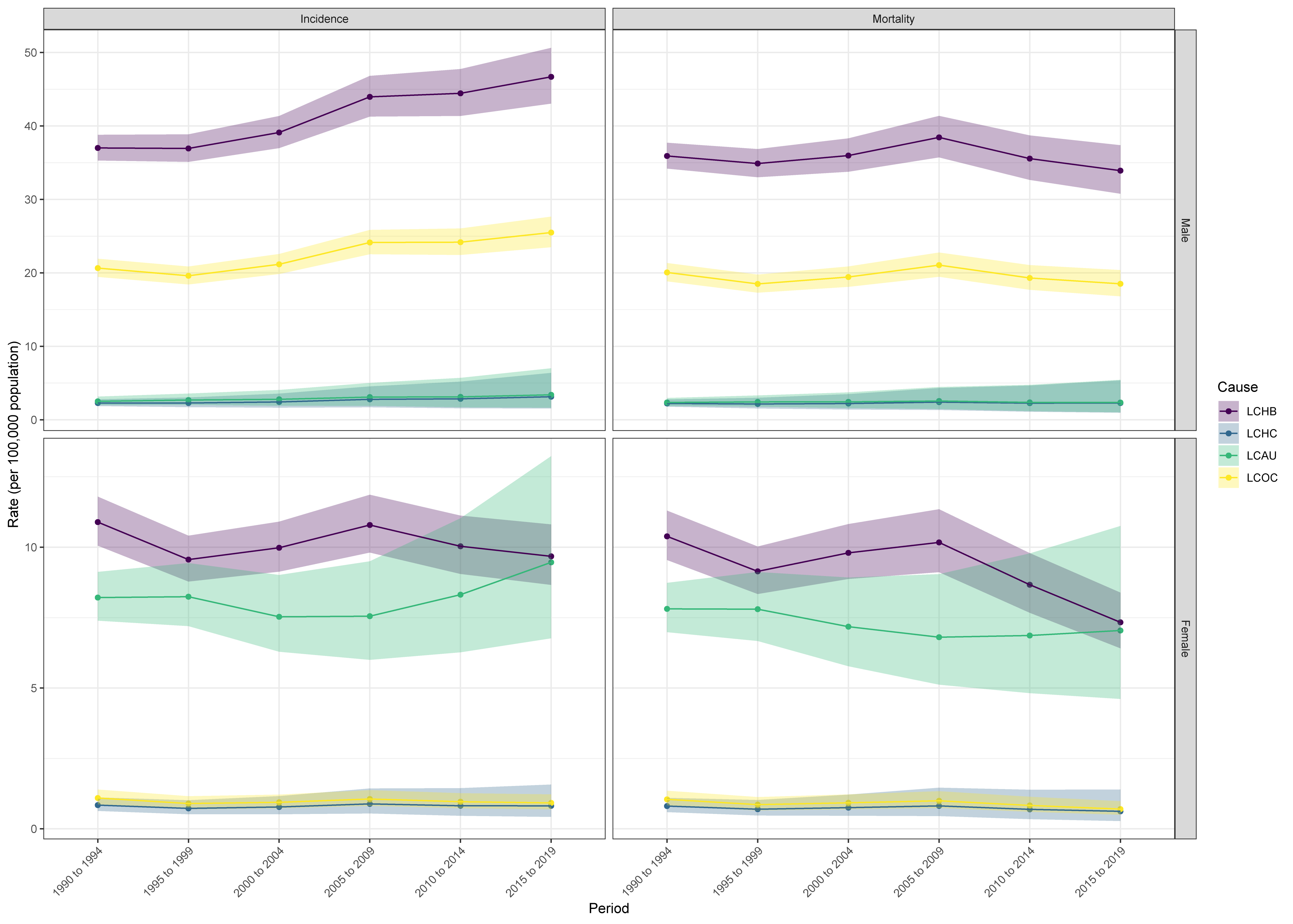


Figure S3. Fitted time trends of liver cancer due to specific etiologies incidence and mortality in males and females in Hubei.
